# Massive Sorghum Collection Genotyped with SSR Markers to Enhance Use of Global Genetic Resources

**DOI:** 10.1371/journal.pone.0059714

**Published:** 2013-04-02

**Authors:** Claire Billot, Punna Ramu, Sophie Bouchet, Jacques Chantereau, Monique Deu, Laetitia Gardes, Jean-Louis Noyer, Jean-François Rami, Ronan Rivallan, Yu Li, Ping Lu, Tianyu Wang, Rolf T. Folkertsma, Elizabeth Arnaud, Hari D. Upadhyaya, Jean-Christophe Glaszmann, C. Thomas Hash

**Affiliations:** 1 Cirad, UMR AGAP, Montpellier, France; 2 International Crops Research Institute for the Semi-Arid Tropics (ICRISAT), Hyderabad, Andhra Pradesh, India; 3 Institute of Crop Science, Chinese Academy of Agricultural Sciences (CAAS), Beijing, China; 4 Bioversity International, Montpellier, France; National Rice Research Center, United States of America

## Abstract

Large *ex situ* collections require approaches for sampling manageable amounts of germplasm for in-depth characterization and use. We present here a large diversity survey in sorghum with 3367 accessions and 41 reference nuclear SSR markers. Of 19 alleles on average per locus, the largest numbers of alleles were concentrated in central and eastern Africa. Cultivated sorghum appeared structured according to geographic regions and race within region. A total of 13 groups of variable size were distinguished. The peripheral groups in western Africa, southern Africa and eastern Asia were the most homogeneous and clearly differentiated. Except for Kafir, there was little correspondence between races and marker-based groups. Bicolor, Caudatum, Durra and Guinea types were each dispersed in three groups or more. Races should therefore better be referred to as morphotypes. Wild and weedy accessions were very diverse and scattered among cultivated samples, reinforcing the idea that large gene-flow exists between the different compartments. Our study provides an entry to global sorghum germplasm collections. Our reference marker kit can serve to aggregate additional studies and enhance international collaboration. We propose a core reference set in order to facilitate integrated phenotyping experiments towards refined functional understanding of sorghum diversity.

## Introduction

Crop domestication is characterised by human selection on wild species for traits useful for food production. This continuous process made possible the development of agriculture and of civilizations. While migrating, man moved together with his crops and spread agriculture worldwide. It led to global development as well as occasional harsh competitions. While many industrial crops have a recent domestication history intermingled with that of colonization, food crops present distributions that have little relation with their domestication place.

Recent global planetary constraints create a new threatening situation; plant breeding is currently faced with unprecedented challenges, which call for global cooperation. Plant genetic resources conceal the matter for future improvement and adaptation. They bear thousands of years of genetic adaptation to multiple conditions and usages by Man. In times when 1) food security is dramatically challenged by population growth, shortage of input supply and climate changes, and 2) genomic tools and methodologies bring about unprecedented capacities of scientific investigation, they are and will remain a stake, matter of competition as well as cooperation.

Sorghum [*Sorghum bicolor* (L.) Moench, 2*n* = 2*x* = 20] is the fifth most important cereal crop in the world. Its use as staple food and fodder confers it the status of a ‘failsafe’ crop in global agro-ecosystems. It is widely adapted to harsh environmental conditions, and more specifically to arid and semi-arid regions of the world. It is currently a model crop for tropical grasses that employ C_4_ photosynthesis because of the availability of its complete genome sequence [1] [2], http://genome.jgi-psf.org/Sorbi1/Sorbi1.info.html).

There are several identified collections of sorghum genetic resources (for example core-collections [3] [4], US converted tropical and breeding lines described in [5], US sweet sorghum collection [6], mutant populations [7], Japanese collection [8], as well as accessions available at ICRISAT). Sorghum’s center of diversity lies in the northeastern quadrant of Africa and it is thought sorghum was domesticated there over 5,000 years before present [9]. Based on spikelet and grain morphology, Harlan and de Wet [9] developed a simplified classification of traditional sorghum cultivars into five basic races: Bicolor (B), Caudatum (C), Durra (D), Guinea (G) and Kafir (K), and ten intermediate races (in all pair-wise combinations of basic races).

Biochemical genetic markers provided the first assessment of neutral genetic variation and enabled demarcation of groups by race and origin [10]. Several generations of DNA-based molecular markers were then used and refined the assessment. In the early 1990 s, restriction fragment length polymorphism (RFLP) markers were effectively utilized for sorghum diversity analysis (reviewed in [11], [12]), genetic mapping (e.g. [13], [14], [15], [16], [17]) and comparative genome mapping ([18], [19], [20], [21]). Later other marker systems were tried, including randomly amplified polymorphic DNAs (RAPDs), simple sequence repeats (SSRs), and amplified fragment length polymorphisms (AFLPs). These markers systems, independently or in combination with others, were efficiently used for sorghum genetic diversity analysis. Diversity array technology (DArT) markers have recently been developed and utilized for genetic diversity assessment and mapping [22], as well as SNPs [23].

SSRs were developed independently by several different research groups (see review by [24], and [25], [26], [27]) and were exploited for genetic diversity analysis. Many of these diversity analyses focused on local collections (e.g. [28], [29], [30], [31]), trait-specific genotypes [e.g. aluminum tolerance [32], sweet stalks ([33], [6]), disease resistance [34]], or a particular race (Guinea [35]).

In front of the large size of the collections available and the diversity of interests expressed in the various studies, we undertook this study in order to provide a better insight into global sorghum genetic diversity and to set a reference, which can attract interest, stimulate cooperation and coordination and enhance interactions and connections among all initiatives. A large collection of sorghum (global composite germplasm collection, GCGC) including over 3300 accessions was thus genotyped with highly polymorphic markers (41 SSRs) providing coverage across all 10 chromosome pairs in the nuclear genome of *Sorghum bicolor*. This was performed in the frame of the Generation Challenge Programme (GCP, www.generationcp.org). It may provide a foundation for more efficient management and utilization of available genetic resources in this crop, as well as a tool for mining alleles of genes controlling important agronomic traits.

## Methods

### Plant Material

Sorghum material studied was mainly selected among ICRISAT's collection ([4]), since ICRISAT has one of the largest crop germplasm collections held in trust by the Consultative Group for International Agricultural Research (CGIAR). ICRISAT's collection includes germplasm of staple food crops of the semi-arid tropics including sorghum, pearl millet, groundnut, pigeonpea, chickpea and several small millets (foxtail millet, finger millet, etc). Chinese material was under-represented in ICRISAT’s collection; so it was complemented with material provided by CAAS. It also included a previously defined core collection, mainly from ICRISAT's collection and extensively studied ([3]). A total of 3367 sorghum accessions were thus studied in this paper, representing cross-compatible sorghum germplasm of broad initial taxonomic status (passport information available in Table S1). This GCP sorghum GCGC included 280 breeding lines and elite cultivars from public sorghum breeding programs, 68 wild and weedy accessions, and over 3000 landrace accessions from collections held by CIRAD or ICRISAT that were selected either from previously defined core collections ([3], [4]), for resistance to various biotic stresses, and/or for variation in other agronomic and quality traits. All three labs, CAAS-China, CIRAD-France and ICRISAT-India, contributed accessions to the study. CIRAD contributed 225 well-characterized genotypes that constitute a mini-core collection representing a very broad range of diversity [11], CAAS contributed 250 accessions comprising sweet sorghums, grain sorghums and glutinous sorghums from China, and the remaining accessions were contributed by ICRISAT. All accessions from this sorghum GCGC collection are publically available, except the 250 provided by CAAS. This collection included representation of all 5 basic races of cultivated sorghum [Bicolor (B), Caudatum (C), Durra (D), Guinea (G) and Kafir (K)] and their ten intermediate collected from different parts of the world (Table 1). All together one third of the accessions were provided by all ten intermediate races (1159 accessions), while the largest numbers of basic races were represented by Durra (651 accessions) and Caudatum (577 accessions).

**Table 1:** Distribution of accessions in the sorghum Global Composite Germplasm Collection (GCGC).

Accession status, race or passport origin	Number of accessions (% of total)
**Status**	
Wild or weedy	68 (2.0%)
Landrace	3013 (89.5%)
Breeding lines or advanced cultivars	280 (8.3%)
Unknown	6 (0.1%)
**Race**	
Bicolor	195 (5.8%)
Caudatum	577 (17.2%)
Durra	656 (19.4%)
Guinea	365 (10.8%)
Kafir	239 (7.1%)
Intermediate	1159 (34.4%)
Unknown	115 (3.3%)
**Passport origin**	
Africa	1926 (57.3%)
Central Africa	224 (6.6%)
Eastern Africa	570 (16.9%)
Southern Africa	735 (21.8%)
Western Africa	397 (11.8%)
Asia	1010 (30.1%)
Eastern Asia	441 (13.1%)
Indian subcontinent	449 (13.3%)
Middle East	120 (3.6%)
North America	227 (6.7%)
Latin America	21 (0.6%)
Unknown	138 (4.0%)
Other	45 (1.3%)

### DNA Extraction

DNA extraction was carried out in the labs contributing the sorghum entries to this study, with a single representative plant providing the DNA for each accession, following a protocol described by [36] for accessions contributed by ICRISAT and as described in [37] for accessions contributed by CIRAD and CAAS. Extracted DNA samples were exchanged between the labs for SSR marker genotyping.

### SSR Markers

All 48 markers used were part of a sorghum SSR kit [24] (http://sorghum.cirad.fr/SSR_kit), which provides reasonable coverage across the sorghum nuclear genome. Marker genotyping at CIRAD was performed on the Genotyping Platform of the Montpellier Languedoc-Roussillon Genopole (GPTR. http://www.gptr-lr-genotypage.com/) for markers *gpsb*067, *gpsb*089, *gpsb*123, *mSbCIR*246, *mSbCIR*262, *mSbCIR*300, *mSbCIR*329, *Sb*5-206 = *Xgap*206, *Sb*6-84 = *Xgap*084, *SbAGB*02, *Xcup*02, *Xcup*14, *Xcup*53, *Xcup*61, *Xcup*63, *Xtxp*010, *Xtxp*015, *Xtxp*040, *Xtxp*057, and *Xtxp*145. The forward primer was designed with a 5′-end M13 extension (5′-CACGACGTTGTAAAACGAC-3′). IRDye® 700 or IRDye®800-labeled PCR products were diluted 10-fold and 4-fold, respectively, and subjected to electrophoresis in 6.5% poly-acrylamide gels with a Licor IR2 system (Licor, USA).

Markers *Xisep*0107, *Xisep*0310, *mSbCIR*223, *mSbCIR*238, *mSbCIR*240, *mSbCIR*248, *mSbCIR*276, *mSbCIR*283, *mSbCIR*286, *mSbCIR*306, *Sb*4-72 = *Xgap*072, *Xcup*11, *Xtxp*012, *Xtxp*021, *Xtxp*114, *Xtxp*136, *Xtxp*141, *Xtxp*265, *Xtxp*273, *Xtxp*278, *Xtxp*320, *Xtxp*321 and *Xtxp*339 were genotyped at ICRISAT. Amplified PCR products, according to their multiplexes, along with internal ROX-400 size standard, were separated by capillary electrophoresis using an ABI 3700 sequencer (Applied Biosystems, USA).

Markers *gpsb*069, *gpsb*148, *gpsb*151, *Xcup*62 and *Xtxp*295, were genotyped at CAAS according to the same protocol used at ICRISAT, except that amplification products, along with ROX-400 size standard, were separated by capillary electrophoresis in single-marker runs.

In all three labs, three control panel DNA samples were used as standard checks ([24], http://sorghum.cirad.fr/SSR_kit), in every PCR and electrophoresis run to facilitate accurate allele calling.

### Data Analysis

SSR markers used in this study showed high reproducibility in PCR amplification and ABI/Licor runs based on the allele sizes produced by control panel entries that were included in every PCR run. SagaGT software (Licor, USA) was used for allele scoring for the markers genotyped at CIRAD. At ICRISAT and CAAS, fragment analysis of PCR products was carried out using GeneScan and Genotyper 3.7 software packages (Applied Biosystems, USA). PCR amplicon sizes were scored in base pairs (bp) based on migration relative to the internal ROX-400 size standard. At ICRISAT these raw allele calls were further processed through the AlleloBin software program (available at http://www.icrisat.org/bt-software-d-allelobin.htm) to provide adjusted allele calls. AlleloBin uses a standard repeat motif length (following the step-wise mutation model [38]) and a least squares algorithm to call allele sizes to integer values as suggested by Idury and Cardon [39], adjusting for imperfections in the co-migration of size standards and PCR products.

Marker data for 7 SSR markers (*gpsb069, gpsp089, gpsb148, gpsb151, Xcup62, Xtxp295* and *Xtxp33*) were removed from the final analysis due to incomplete data or low quality genotyping. Finally, 3367 accessions were retained for further analysis across 41 markers (Table 1).

Data files were assembled in a database (Sagacity v.10, Rami, in preparation) and allele sizes were checked for congruency and adjusted according to the allelic references provided in the SSR kit [24].

Descriptors of observed genetic diversity, such as allele number per marker, observed heterozygosity (*Ho*) and gene diversity (expected heterozygosity, *He*) were calculated using PowerMarker v3.25 software [40]. Allelic richness and private alleles by locus were estimated using ADZE software [41]. Genetic distance between groups, estimated by *F*_st_ statistics, was calculated with hierfstat R package [42]. Mann-Whitney (MW) tests were used to determine whether estimates were significantly different between groups.

To identify the pair-wise genetic relationships between the accessions of this sorghum global composite germplasm collection, a genetic dissimilarity matrix was calculated using simple matching with DARwin v5 software [43] (available at http://darwin.cirad.fr/darwin/Home.php). An overall representation of the diversity structure was obtained by a factorial analysis using the distance matrix, while individual relations were analyzed with a tree construction based on Neighbor Joining (NJ) method, as implemented in DARwin v5.

In order to test for sample clustering in conjunction with admixture between sub-groups, Bayesian statistics based on Monte Carlo Markov Chain algorithm were used. Although the Instruct software package [44] was developed to handle specifically species with a high level of inbreeding, as expected for sorghum, it was not used here because it cannot handle such a large number of samples. STRUCTURE software v.2.3.3 [45] was thus preferred. One hundred replicates were performed for each K, the number of clusters considered. Each run used a burn-in period of 100,000 iterations followed by 200,000 iterations. For each K, the 10 runs presenting the highest maximum likelihood value were kept, and sample assignation to groups was performed with CLUMPP software (up to K = 6, greedy algorithm, 1000 repeats, over K = 6, large K greedy algorithm, 1000 permutations) in order to deal with label switching or multimodalities. Estimate of the best cluster number was performed following [46] with a R (http://www.r-project.org/) script modified from [47]. It was compared to information given by each cluster, and identified when no new individual presented a majority of ancestry in a new cluster (threshold 0.7). Genome plot representations were performed using a specifically developed R script (available upon request).

A Reference Set of 383 sorghum accessions including *S. bicolor* subspecies *bicolor* and wild *S. bicolor* subspecies *verticilliflorum* was chosen among the publically available accessions to best represent genetic diversity as well as geographic origins. Maximum Length Subtree function of DARwin v5 software [43] was used to deal with genetic diversity. It is based on successive elimination of samples, each eliminated sample presenting a minimal reduction of overall diversity, measured as branch length of a tree. Since in the GCGC collections, phenotyping data were already available on a subset of diverse accessions ([11], [4]), this subset was first analyzed to reduce redundancy. Widely used breeding lines completed it. A first run of completion of these accessions was performed on *S. bicolor* only, checking that all geographic origins are conserved. The same process was performed for wild accessions, and both datasets were merged to represent the Sorghum Reference Set.

## Results

### Global Variation

#### Level of polymorphism

All 41 SSR markers used detected polymorphism in the sorghum GCGC. A total of 783 SSR marker alleles were detected, with an average of 19.2 alleles per marker. Numbers of alleles per marker ranged from three (*Xtxp*136) to 39 (*SbAGB*02), with an average of 3.44% of missing data (Table 2).

**Table 2:** Marker characteristics and genetic diversity of the sorghum Global Composite Germplasm Collection (GCGC).

SSRmarker	Forward primer sequence(5'-3')	Reverse primer sequence(5'-3')	Repeat	Chr	AlleleNumber	Genediversity(*He*)	Observedheterozygosity(*Ho*)
**gpsb067**	TAGTCCATACACCTTTCA	TCTCTCACACACATTCTTC	(GT)10	8	15	0.681	0.032
**gpsb123**	ATAGATGTTGACGAAGCA	GTGGTATGGGACTGGA	(CA)7+(GA)5	8	14	0.720	0.030
**mSbCIR223**	CGTTCCAATGACTTTTCTTC	GCCAATGTGGTGTGATAAAT	(AC)6	2	10	0.703	0.023
**mSbCIR238**	AGAAGAAAAGGGGTAAGAGC	CGAGAAACAATTACATGAACC	(AC)26	2	27	0.859	0.027
**mSbCIR240**	GTTCTTGGCCCTACTGAAT	TCACCTGTAACCCTGTCTTC	(TG)9	8	35	0.746	0.034
**mSbCIR246**	TTTTGTTGCACTTTTGAGC	GATGATAGCGACCACAAATC	(CA)7.5	7	13	0.237	0.010
**mSbCIR248**	GTTGGTCAGTGGTGGATAAA	ACTCCCATGTGCTGAATCT	(GT)7.5	5	13	0.659	0.226
**mSbCIR262**	GCACCAAAATCAGCGTCT	CCATTTACCCGTGGATTAGT	(CATG)3.25	10	30	0.663	0.044
**mSbCIR276**	CCCCAATCTAACTATTTGGT	GAGGCTGAGATGCTCTGT	(AC)9	3	10	0.559	0.023
**mSbCIR283**	TCCCTTCTGAGCTTGTAAAT	CAAGTCACTACCAAATGCAC	(CT)8 (GT)8.5	10	24	0.810	0.020
**mSbCIR286**	GCTTCTATACTCCCCTCCAC	TTTATGGTAGGATGCTCTGC	(AC)9	1	19	0.795	0.026
**mSbCIR300**	TTGAGAGCGGCGAGGTAA	AAAAGCCCAAGTCTCAGTGCTA	(GT)9	7	11	0.689	0.031
**mSbCIR306**	ATACTCTCGTACTCGGCTCA	GCCACTCTTTACTTTTCTTCTG	(GT)7	1	5	0.616	0.015
**mSbCIR329**	GCAGAACATCACTCAAAGAA	TACCTAAGGCAGGGATTG	(AC)8.5	5	13	0.746	0.028
**Sb4-72**	TGCCACCACTCTGGAAAAGGCTA	CTGAGGACTGCCCCAAATGTAGG	(AG)16	6	24	0.699	0.021
**Sb5-206**	ATTCATCATCCTCATCCTCGTAGAA	AAAAACCAACCCGACCCACTC	(AC)13/(AG)20	9	34	0.941	0.046
**Sb6-84**	CGCTCTCGGGATGAATGA	TAACGGACCACTAACAAATGATT	(AG)14	2	32	0.859	0.027
**SbAGB02**	CTCTGATATGTCGTTGTGCT	ATAGAGAGGATAGCTTATAGCTCA	(AG)35	7	39	0.668	0.033
**Xcup02**	GACGCAGCTTTGCTCCTATC	GTCCAACCAACCCACGTATC	(GCA)6	9	10	0.656	0.030
**Xcup11**	TACCGCCATGTCATCATCAG	CGTATCGCAAGCTGTGTTTG	(GCTA)4	3	6	0.501	0.050
**Xcup14**	TACATCACAGCAGGGACAGG	CTGGAAAGCCGAGCAGTATG	(AG)10	3	22	0.542	0.019
**Xcup53**	GCAGGAGTATAGGCAGAGGC	CGACATGACAAGCTCAAACG	(TTTA)5	1	11	0.577	0.021
**Xcup61**	TTAGCATGTCCACCACAACC	AAAGCAACTCGTCTGATCCC	(CAG)7	3	6	0.474	0.024
**Xcup63**	GTAAAGGGCAAGGCAACAAG	GCCCTACAAAATCTGCAAGC	(GGATGC)4	2	11	0.316	0.033
**Xisep0107**	GCCGTAACAGAGAAGGATGG	TTTCCGCTACCTCAAAAACC	(TGG)4	3	5	0.556	0.014
**Xisep0310**	TGCCTTGTGCCTTGTTTATCT	GGATCGATGCCTATCTCGTC	(CCAAT)4	2	10	0.252	0.019
**Xtxp10**	ATACTATCAAGAGGGGAGC	AGTACTAGCCACACGTCAC	(CT)14	9	15	0.778	0.055
**Xtxp12**	AGATCTGGCGGCAACG	AGTCACCCATCGATCATC	(CT)22	4	30	0.935	0.039
**Xtxp15**	CACAAACACTAGTGCCTTATC	CATAGACACCTAGGCCATC	(TC)16	5	23	0.863	0.062
**Xtxp21**	GAGCTGCCATAGATTTGGTCG	ACCTCGTCCCACCTTTGTTG	(AG)18	4	33	0.625	0.036
**Xtxp40**	CAGCAACTTGCACTTGTC	GGGAGCAATTTGGCACTAG	(GGA)7	7	21	0.380	0.021
**Xtxp57**	GGAACTTTTGACGGGTAGTGC	CGATCGTGATGTCCCAATC	(GT)21	6	29	0.823	0.058
**Xtxp114**	CGTCTTCTACCGCGTCCT	CATAATCCCACTCAACAATCC	(AGG)8	3	11	0.597	0.040
**Xtxp136**	GCGAATAGCATCTTACAACA	ACTGATCATTGGCAGGAC	(GCA)5	5	3	0.457	0.022
**Xtxp141**	TGTATGGCCTAGCTTATCT	CAACAAGCCAACCTAAA	(GA)23	10	22	0.887	0.035
**Xtxp145**	GTTCCTCCTGCCATTACT	CTTCCGCACATCCAC	(AG)22	6	32	0.917	0.055
**Xtxp265**	GTCTACAGGCGTGCAAATAAAA	TTACCATGCTACCCCTAAAAGTGG	(GAA)19	6	26	0.919	0.058
**Xtxp273**	GTACCCATTTAAATTGTTTGCAGTAG	CAGAGGAGGAGGAAGAGAAGG	(TTG)20	8	21	0.689	0.030
**Xtxp278**	GGGTTTCAACTCTAGCCTACCGAACTTCCT	ATGCCTCATCATGGTTCGTTTTGCTT	(TTG)12	7	25	0.474	0.027
**Xtxp320**	TAAACTAGACCATATACTGCCATGATAA	GTGCAAATAAGGGCTAGAGTGTT	(AAG)20	1	19	0.847	0.046
**Xtxp321**	TAACCCAAGCCTGAGCATAAGA	CCCATTCACACATGAGACGAG	(GT)4+(AT)6+(CT)21	8	30	0.934	0.033
				Mean	**19.244**	**0.674**	**0.037**
				Min	**3**	**0.237**	**0.010**
				Max	**39**	**0.941**	**0.226**

Na: Number of alleles, He: unbiased genetic diversity, according to Nei (1987), Ho: observed heterozygosity.

Availability of marker data ranged from 88% (*gpsb*123) to 99% (*Xcup*63). On average 3.44% of data was missing.

A mean gene diversity (expected heterozygosity, *He*) of 0.67 was observed across the sorghum global composite collection, with values ranging from 0.24 (*mSbCIR*246) to 0.94 (*Sb*5-206) for individual markers (Table 2). Even though *SbAGB*02 produced the highest number of alleles (39), it presented an intermediate *He* value of 0.67 because 92% of these alleles can be considered as rare (74% below 1% frequency). With the exception of *mSbCIR*248, which had an unusually high observed heterozygosity (*Ho*) value of 0.23, the *Ho* values ranged from 0.01 (*mSbCIR*246) to 0.06 (*Xtxp015*) with a mean of 0.03. Its outstanding *Ho* value suggests that marker *mSbCIR*248 may have detected more than one polymorphic locus, but this is not confirmed yet by *in-silico* hybridisation to the complete reference sorghum sequence.

#### Allelic distributions among taxonomic components

Allele number distribution and genetic diversity in sorghum GCGC according to biological status, race, and geographic origin is reported in Table 3. All 41 SSR markers used detected polymorphism in all compartments. The 3013 landrace accessions (87% of total accessions) contributed 94% of SSR marker alleles detected, all breeding lines (including advanced cultivars, 280 accessions, 8%) and wild and weedy accessions (68 entries, 2%) captured 57% and 65% of the detected alleles, respectively. Allelic richness of standardized sample sizes of 100 haploid genomes showed that breeding lines tended to present less genetic diversity compared to landraces and wild samples, and that wild samples appeared more diverse (MW test, non-significant P values, P = 0.15 for breeding-landrace comparison and P = 0.08 for landrace-wild comparison). This is confirmed for private alleles (MW test, P<0.05 and P<0.01, respectively), with three times more private allele numbers in wild and weedy samples than in landraces (3.25 vs 1.04) and larger average expected heterozygosity values (MW test, P = 0.017).

**Table 3:** Genetic diversity in the sorghum Global Composite Germplasm Collection (GCGC) and in the Reference Set, partitioned into biological status, races and geographic origins as indicated in passport data.

	Global Composite Germplasm Collection							Reference Set				
	N	Na	MeanNa	Arich(100)	PrivA(100)	GeneDiversity	*Ho*	N	Nall	MeanNa	GeneDiversity	*Ho*
**Overall**	**3367**	**783**	**19.10**	**10.04** **(0.95)** 1		**0.674**	**0.037**	**383**	**613**	**14.95**	**0.712**	**0.048**
**Status**												
Wild or weedy	68	508	12.39	11.91(0.95)	3.25 (0.41)	0.743	0.234	23	355	8.66	0.748	0.216
Landrace	3013	736	17.95	10.06(0.95)	1.04 (0.17)	0.671	0.032	332	576	14.05	0.707	0.035
Breeding lines oradvanced cultivars	280	443	10.80	8.53 (0.78)	0.58 (0.09)	0.630	0.042	28	263	6.41	0.621	0.058
Unknown	6	163	3.98			0.536	0.153	0	0	0	0.000	0.000
**Race**												
Bicolor	195	483	11.78	9.65 (0.92)	0.74 (0.10)	0.669	0.041	36	334	8.15	0.695	0.045
Caudatum	577	539	13.15	9.16 (0.94)	0.59 (0.18)	0.626	0.029	76	378	9.22	0.633	0.040
Durra	656	521	12.71	8.91 (0.88)	0.47 (0.07)	0.600	0.043	44	312	7.61	0.655	0.024
Guinea	365	476	11.61	8.62 (0.80)	0.65 (0.15)	0.628	0.025	64	331	8.07	0.661	0.027
Kafir	239	327	7.98	5.97 (0.59)	0.15 (0.04)	0.410	0.021	23	191	4.66	0.444	0.031
Intermediate	1159	629	15.34	9.78 (0.94)	0.48 (0.06)	0.661	0.029	104	450	10.98	0.703	0.039
Unknown	116	376	9.17			0.610	0.085	18	236	5.76	0.626	0.100
**Passport origin**												
Africa	1853	680	16.59	9.83 (0.91)	1.27 (0.18)	0.654	0.032	257	558	13.61	0.697	0.040
Central Africa	219	444	10.83	8.68 (0.85)	0.66 (0.15)	0.630	0.037	35	281	6.85	0.645	0.040
Eastern Africa	537	571	13.93	9.86 (0.98)	1.06 (0.15)	0.670	0.036	85	431	10.51	0.688	0.046
Southern Africa	718	508	12.39	7.49 (0.72)	0.59 (0.10)	0.511	0.026	74	372	9.07	0.592	0.038
Western Africa	379	512	12.49	8.82 (0.71)	0.99 (0.14)	0.611	0.038	63	351	8.56	0.674	0.034
Asia	976	594	14.49	8.91 (0.89)	1.12 (0.19)	0.587	0.043	71	372	9.07	0.644	0.048
Eastern Asia	439	438	10.68	7.49 (0.81)	1.05 (0.22)	0.474	0.052	18	181	4.41	0.466	0.027
Indian Subcontinent	417	454	11.07	7.98 (0.77)	1.16 (0.15)	0.576	0.022	35	278	6.78	0.623	0.030
Middle East	120	400	9.76	8.64 (0.82)	1.82 (0.28)	0.602	0.085	18	238	5.8	0.614	0.106
Europe	1	42	1.02			–	–	1	42	1.02	–	–
Mediterranean Basin	29	271	6.61			0.637	0.031	7	161	3.93	0.649	0.037
North America	185	506	12.34	10.10 (0.91)	1.51 (0.18)	0.690	0.042	34	330	8.05	0.710	0.093
South America	21	201	4.90			0.587	0.038	0	0	0	0.000	0.000
Australia	13	166	4.05			0.486	0.038	2	82	2	0.396	0.195
Unknown	3	93	2.27			0.311	0.008	0	0	0	0.000	0.000

1 calculated over 383 diploid genomes.

Partition into geographic origins is limited to landraces and wild samples for which geographic origin relates to reality.

N: number of accessions; Na: total number of alleles; MeanNa: mean number of alleles per marker, ARich: allelic richness calculated according to Petit *et al.* (1998) as in [41] for standard sample sizes of 100 genomes; PrivA: Private allele number per marker calculated according to [41] for standard sample sizes of 100 genomes (allelic richness and private allele number per marker were calculated for those classes which presented more than 100 genomes, by continent – Africa, Asia and Northern America – and in each sub-continent – Africa and Asia –), before the pipe are the values observed inside the continent, each continent being analyzed separately, after the pipe are the values observed when all 3 continents are analyzed together at the sub-continent level; *Ho*: observed heterozygosity.

Except of Kafir, the other four basic races exhibited no significant difference in allele numbers per marker. Kafir presented the smallest numbers of alleles per marker and private alleles (almost 3 alleles per marker less than the four others, MW tests, P<0.001) and a lower genetic diversity (*He* = 0.41 versus *He* of 0.60–0.67 for the other four basic races). The Guinea race encompassed the Guinea margaritiferum (Gma) accessions (at least 12), for which two markers (*mSbCIR*240 and *Xcup*53) were found to be monomorphic, whereas allelic richness of same sample sizes of all races, including other Guineas, ranged 1.58–7.02 and 1.56–3.04, respectively.

Highest numbers of alleles 680 (86.8%) were detected among the accessions of African origin. When correcting for sample sizes at the continent level, North American accessions (all originally introduced from elsewhere, or derived from such introduced materials) tended to be more diverse both in terms of total numbers and private alleles, but the MW tests were not conclusive. In Africa, Eastern Africa exhibited the largest gene diversity, followed by Central Africa while Southern Africa was the poorest (MW test, P = 0.02). In Asia, Middle East origins presented a higher genetic diversity than India and East Asia (MW test, private alleles, P = 0.05).

#### Allele specificity

Among the 783 alleles detected, 35% (280) were observed only in cultivated sorghum accessions and 5% (40) only in wild/weedy accessions.

Among the 41 SSR markers analyzed, 17 markers produced alleles unique to wild/weedy accessions, three (*mSbCIR*276, *Xisep*0107 and *Xtxp*136) for cultivated accessions, and *Xisep*0310 did not detect alleles unique to either the cultivated or wild/weedy accessions. Among these 17 SSRs, eight markers (*gpsb*067, *gpsb*123, *mSbCIR*223, *mSbCIR*238, *Sb*5-206, *Xcup*02, *Xcup*53 and *Xtxp*265) detected only one allele unique to wild/weedy accessions and a maximum of six such alleles were detected for marker *Xtxp*273. Out of the 68 wild/weedy accessions included in this study, 37 accessions produced these 40 alleles that were not detected in cultivated accessions. Wild accession IS 18931 alone contributed six alleles that were not found among the cultivated accessions and IS 18818 (of the *aethiopicuum* group within *S. bicolor* subspecies *verticilliflorum*) contributed five such alleles. Three alleles that were not detected among the cultivated accessions were detected in the only accession of *S. propinquum* (IS 18933) included in this global composite germplasm collection. Among the 3299 cultivated accessions, 40 of 41 SSR markers detected alleles not found among the 68 wild/weedy accessions. This is probably related to sample sizes differences and to the fact that SSR markers used in this study were chosen for their genome-wide distribution, based on existing maps built from crosses of cultivated accessions only, representing thus a diversity compartment different from wild/weedy entries.

The largest number of alleles unique to cultivated accessions was detected for *mSbCIR*240, for which 24 out of 35 alleles detected in the global collection were detected only in cultivated accessions, but no alleles of this marker were detected only in wild/weedy accessions. The overall frequency of rare marker alleles in the sorghum GCGC was very high. Across the 3367 accessions, 428 rare alleles (54.2%) below 1% frequency and 621 rare alleles (78.7%) below 5% frequency were detected.

### Patterns of Multi-locus Diversity

#### Factorial analysis

**Figure 1:**
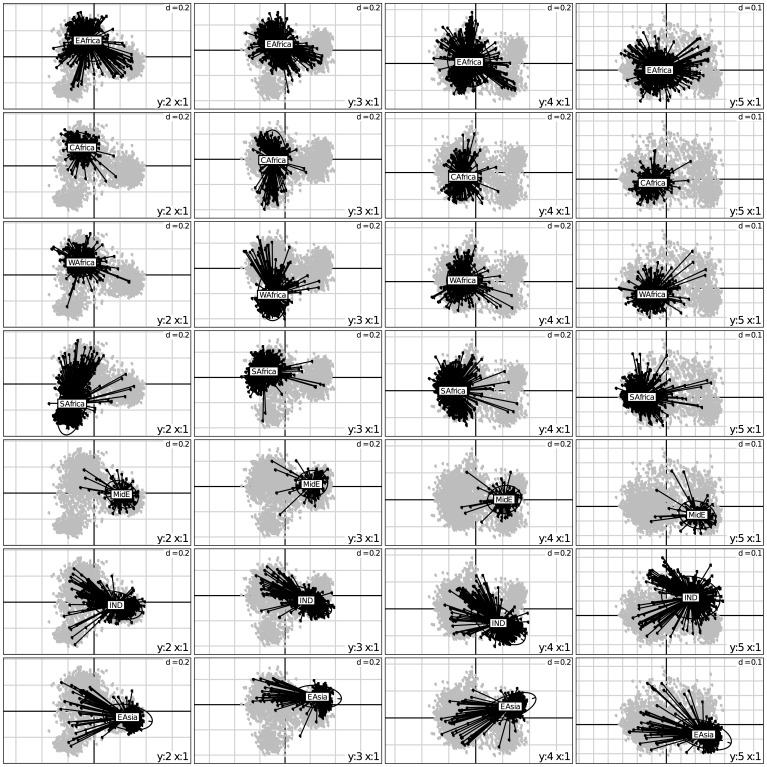
Factorial analysis of the simple matching distance matrix. Representation of the first four axes with accessions characterized by the seven main geographic origins. EAfrica: Eastern Africa, CAfrica: Central Africa, WAfrica: Western Africa, MidE: Middle East countries, IND: Indian subcontinent, EAsia: Eastern Asia, SAfrica: Southern Africa.

**Figure 2:**
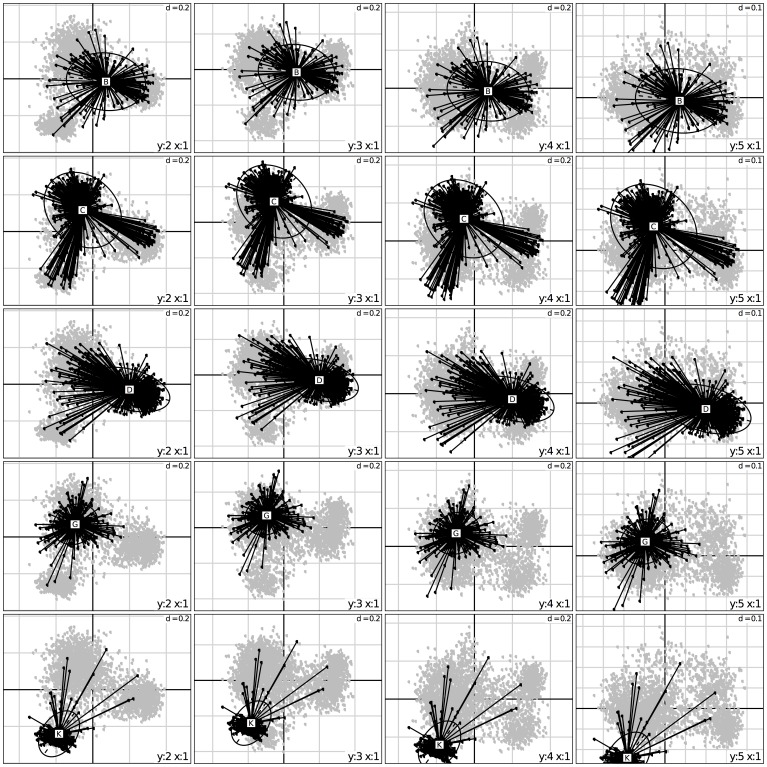
Factorial analysis of the simple matching distance matrix. Representation of the first four axes with accessions characterized by races. B: Bicolor, C: Caudatum, D: Durra, G: Guinea, K: Kafir. Based on allelic richness, there is a trend for Bicolor to be more diverse, followed by Caudatum, Durra, Guinea and finally Kafir being significantly less diverse.

Factorial analysis (FA) of the SSR-based dissimilarity matrix of the complete sorghum GCGC (3367 accessions) showed that the first four axes were to be considered (See plot in Figure S1). The first axis enabled the separation of accessions collected in Africa versus more eastern origins (including some of eastern Africa) (6.05% of the global inertia) (Figure 1). The second (4.09%) and third axes (2.92%) refined the situation of Africa by separating southern Africa and western Africa from central and eastern Africa. Finally the fourth axis (2.35%) enabled the separation of origins from the Indian subcontinent, the Middle East, and eastern Asia. The reference to the racial classification (Figure 2) yields a much less coherent picture, with most races distributed over the whole planes of the FA, with the sole exception of Kafir, clearly separated on plane (1, 2).

#### Classification using bayesian assignations (STRUCTURE)

**Figure 3:**
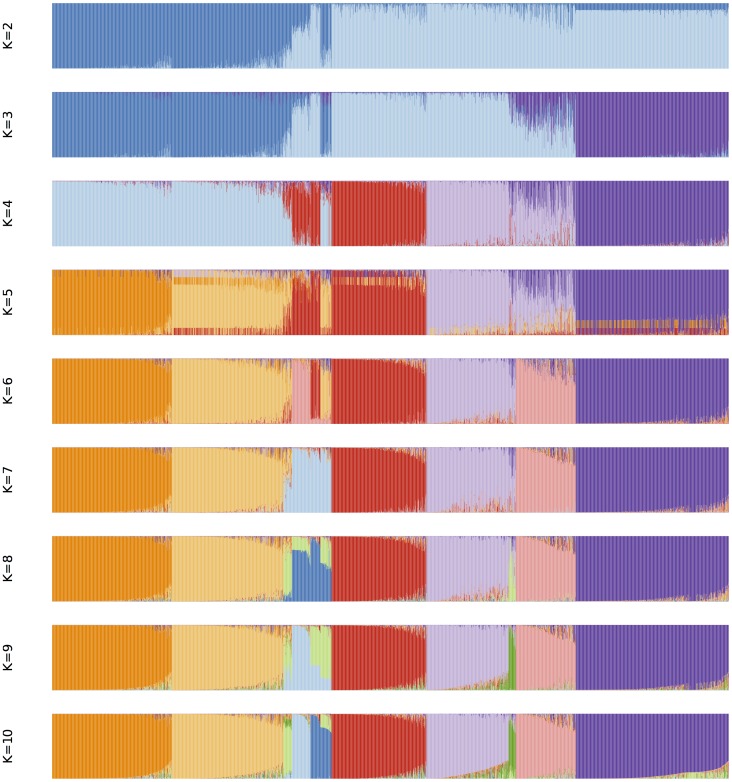
Genome representation of the sorghum GCGC collection, obtained from the assignation by STRUCTURE software at a 0.7 threshold (Pritchard et al. 2000) of each sample in K hypothetical sub-groups. In this study, K varied from 2 (top) to 10 (bottom). Each accession on the X-axis is represented by K colours ordered according to a decreasing genome fraction on the Y-axis. At K = 10, Group 1 in orange: C, CB and D from Eastern Asia, Group 2 in light orange: D and B from the Indian subcontinent, Group 3 in light green: D from Eastern Africa, Group 4 in light blue: B and DB from Eastern Africa, Group 5 in dark blue: G and Gma from Western Africa and B from North America, Group 6 in red: D, DC and G from Western Africa, Group 7 in light purple: C from Central and Eastern Africa, Group 8 in dark green: C and GC from Southern Africa, Group 9 in pink: G from Asia and Southern Africa and C from Eastern Africa, and Group 10 in purple: GC, K and KC from Southern Africa.

Bayesian assignations to sub-groups were performed for 2 to 10 populations (Figure 3), after which no new group was detected with an admixture threshold of 0.7. Two-thirds (2190) of the accessions could be assigned to one of these 10 groups. The unassigned accessions presented genomes scattered among different groups. They included 85% of the wild (58), 54% of the breeding accessions (151), half of the intermediate or unknown races (552), half of the Caudatum (265) and one fifth of the Durra (142) mainly from Eastern Africa. Analysis of assignation rate showed, however, that accessions could be grouped primarily in three populations followed by a sub-division into seven populations (Figure S2).

The first three subdivisions obtained by Bayesian assignment (Figure 3, K = 2, 3 and 4) reflected the main features revealed by the first three axes of the FA and the fourth subdivision (Figure 3, K = 5) reflected axis 4. The subsequent subdivisions also corroborated patterns that appeared through the FA. Thus, Group 1 included Caudatum, Caudatum-Bicolor and Durra from Eastern Asia; Group 2 encompassed Durra and Bicolor from the Indian subcontinent, while Group 3 exhibited Durra from Eastern Africa. Bicolor and Durra-Bicolor from Eastern Africa were assigned in Group 4. Group 5 included Guinea and Guinea margaritiferum from Western Africa and Bicolor from North America. Group 6 appeared as a well-separated group made predominantly of Guinea accessions from western Africa, accompanied by intermediate race Durra-Caudatum materials from western Africa while Group 7 was made essentially of materials collected from eastern Africa and central Africa generally classified as race Caudatum (visible along FA axis 3). Group 8 was a small and heterogeneous group made of Durra and Caudatum race accessions from central Africa. Group 9 was made essentially of Guinea race accessions from the Indian subcontinent and southern/eastern Africa with Guinea-Caudatum (GC) intermediate race accessions from various parts of Africa. Group 10 was made almost exclusively of accessions from southern Africa of race Kafir or intermediate race Kafir-Caudatum (KC).

#### Neighbor joining analysis

**Figure 4:**
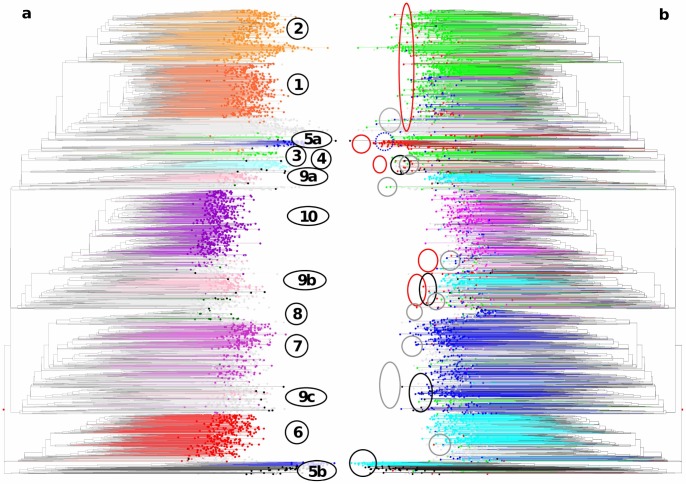
Hierarchical NJ cluster analysis of 3367 sorghum accessions of a global composite germplasm collection based on allelic data from 41 SSR markers (simple matching distance). a- Accessions grouped by Bayesian analysis (Figure 3, K = 10) are represented in color, corresponding to Group 1 in orange, Group 2 in light orange, Group 3 in light green, Group 4 in light blue, Group 5 in dark blue, Group 6 in red, Group 7 in light purple, Group 8 in dark green, Group 9 in pink, and Group 10 in purple. NJ clustering enabled finer resolution of these groups, leading to subdivisions into Group 5a and Group 5b in dark blue, Group 9a, Group 9b and Group 9c in pink. Unassigned accessions are presented in grey. Wild accessions are presented in black. b- Accessions coloured according to their classification in various taxonomic components: Bicolor in red, Caudatum in dark blue, Durra in green, Guinea in light blue, Kafir in purple, unclassified in grey, wild in black. The dendrogram sectors including dispersed components accessions (wild/weedy, Bicolor, and unclassified) are highlighted by circles of the corresponding colours (black, red, grey).

The NJ dendrogram representation on all samples revealed global congruence with the Bayesian assignment with a few apparent discrepancies (Figure 4).

The main discrepancies were the splits of Group 5 and Group 9 into distinct dendrogram sectors. Within Group 5, this split corresponded well with a Bicolor vs Guinea differentiation and led to the distinction of 5a and 5b. Group 9 split into three components 9a, 9b and 9c, 9a and 9b being essentially made of Guinea varieties from South Asia and eastern and southern Africa, respectively, and 9c made of a few Caudatum varieties from eastern Africa. The NJ analysis also threw light on an array of unclassified accessions in the periphery of groups 1 and 2, consisting predominantly of Durra and DC accessions from the Middle East. Group 3 was also challenged by the NJ representation, with most accessions in one dendrogram sector but several of them in another; the size of this group was, however, too small for justifying internal sub-divisions.

#### Distribution of taxonomic components

The classification derived from the STRUCTURE analysis complemented by the NJ dendrogram enabled analyzing the distribution of the various *a priori* taxonomic components. The NJ dendrogram further helped locating all the unassigned materials in relation to the groups that it supported or revealed.

Wild and weedy sorghum accessions were mainly found in four dendrogram sectors (Figure 4). Almost two-thirds (40) of accessions of *S*. *bicolor* subspecies *verticilliflorum* (belonging to races *aethiopicuum*, *arundinaceum*, *verticilliflorum*, and *virgatum*) of diverse origins, as well as weedy intermediate *S*. *bicolor* subspecies *drummondii* clustered around Group 5b. A separate group of *drummondii* and *verticilliflorum* accessions from eastern Africa was observed around Group 9c, associated with cultivated materials from Sudan and Uganda. Another group of *drummondii* accessions from Tanzania, Kenya and Zimbabwe were clustered around Group 9b materials from southern and eastern Africa. Finally, a group of wild and weedy accessions from eastern Africa clustered around Group 4 in close proximity to intermediate race Durra-Bicolor accessions from that region.

The 195 accessions classified as race Bicolor were scattered across many dendrogram sectors and no distinct Bicolor cluster was observed, other than Group 5a, comprised of accessions specifically collected to represent “broom sorghum”. However, the periphery of Groups 1, 2, 4, 5b, 9a and 10 appeared Bicolor-enriched. The four Bicolor accessions close to Group 5b fell among wild/weedy accessions.

Guinea accessions were mainly grouped into four separate dendrogram sections (Figures 3 and 4). Some Guinea accessions, mainly *roxburghii* sub-race materials from the Indian subcontinent and southern Africa, were in Group 9a. A large number of Guinea race accessions from southern Africa (mainly of the *conspicuum* and *roxburghii* sub-race materials from Tanzania and Malawi) were clustered in Group 9b. Another large cluster of Guinea race accessions, mainly from western Africa (Mali, Ghana, Nigeria, Burkina Faso, etc.) and including sub-races *gambicum* and *guineense,* were found in Group 6. Accessions of the *margaritiferum* (Gma) sub-race from western Africa formed a separate Group 5b in close association with wild and weedy accessions.

Caudatum race accessions (577) were broadly dispersed. The vast majority originated from eastern Africa and grouped in and around Groups 7 and 9c. The others followed a geographic organization, with accessions from China in Group 1 and accessions from western Africa and southern Africa in Groups 6 and 10, respectively.

The Durra race was the most widely represented in the GCGC (656 accessions). Most were distributed across several major clusters, with a strong geographical organization. Most Durra accessions from the Indian subcontinent were in Group 2 along with related intermediate materials from that region. Accessions from eastern Asia (mostly from China) were found in Group 1 and accessions from the Middle East and eastern Africa fell in the components of Group 3, while smaller numbers of Durra accessions were in the periphery of Groups 6, 7 and 9c. Interestingly, five Durra accessions clustered with wild/weedy accessions in the vicinity of Group 5b.

The Kafir accessions (239) were mostly from southern Africa and fell in Group 10, together with Kafir-Caudatum and Kafir-Durra accessions from the same region.

The majority of intermediate race accessions were grouped according to their geographic origin. Guinea-Caudatum (GC) was the most common (361 accessions) and was scattered across all NJ sectors, with a majority in the vicinity of Group 7. Durra-Caudatum (DC) was the next most common intermediate race (330 accessions), and was geographically distributed around Group 6 (western Africa) and around Groups 1 and 2 (Mediterranean Basin and the Middle East). Caudatum-Bicolor (CB) accessions were predominantly from eastern Asia and fell in and around Group 1 whereas Durra-Bicolor (DB) accessions from the Indian subcontinent and eastern Africa fell in and around Groups 2 and 4, respectively. Ten intermediate race accessions grouped with wild/weedy accessions close to Group 5b.

A total 430 trait-specific accessions were included in the sorghum GCGC. Many of them were classified as race Caudatum, including accessions resistant to downy mildew, which were clustered according to their origins in Groups 2, 6, 7 and 9c. Stem borer resistant genotypes of race Durra from the Indian subcontinent and Africa were grouped together in Group 2. Genotypes with the capacity to germinate through crusted soil were found in various groups in accordance with their origin and race. Most midge resistant genotypes were found in Group 7. Most of the sweet stalk sorghums that are of increasing interest globally were observed to have Caudatum race background and fell into Group 7. Broom sorghum accessions of race Bicolor from USA formed a specific single Group 5a, whereas all pop sorghum accessions belonging to race Guinea from the Indian subcontinent grouped together in Group 9a. The latter two groups are both small in size and might actually exist because of an over-representation of specialty sorghums gathered for a targeted purpose and resting on a narrow genetic basis.

### Global Differentiation Pattern

**Figure 5:**
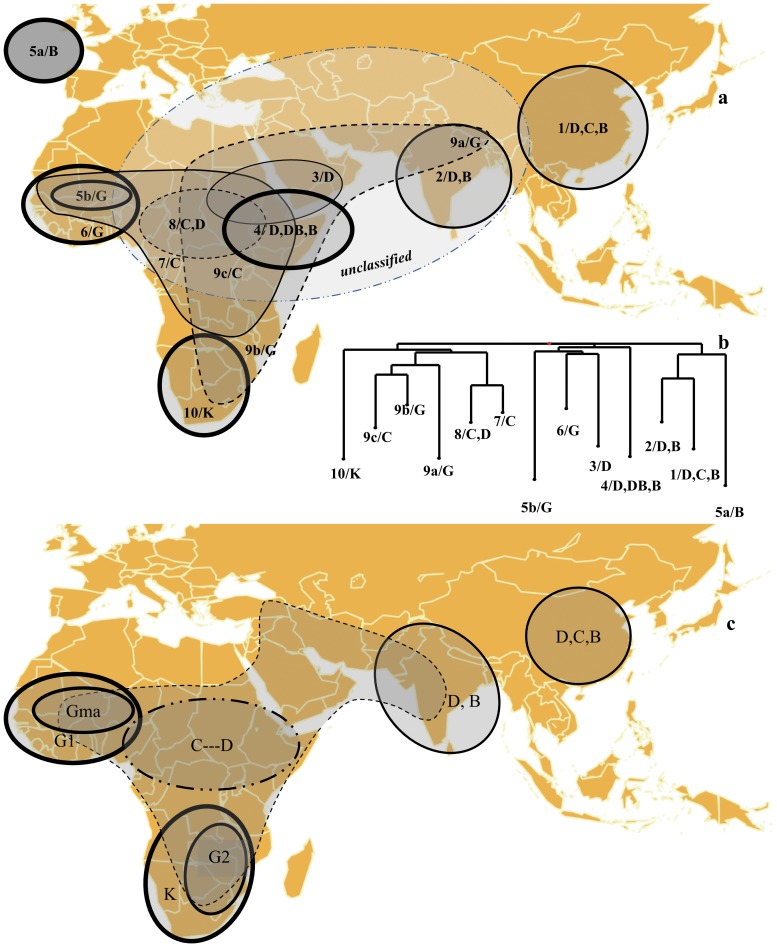
Schematic representation of the pattern of diversity in sorghum projected on a geographical map. a- The groups as identified in Figure 4 are drawn according to their geographical distribution and the predominant race(s) is (are) indicated. The groups are framed differently to reflect their higher (thicker frame) or lower (thin, dotted frame) levels of differentiation as estimated through the *F*_ST_ parameter and the distribution of intermediates. Group 5a actually originates from a collection in USA. b- NJ dendrogram of *F*_ST_ distances between groups identified as in Figure 4. c- Pure races and main regions are predominantly featured, but the intermediate types or regions fall in continuity with this landscape (dotted lines). Races are framed differently to reflect their higher (thicker frame) or lower (thin, dotted frame) levels of differentiation as estimated through the *F*_ST_ parameter as in Figure 5a.

The differentiation between all the components derived from the confrontation of both classification methods was assessed using the *F*_ST_ estimate (Table S2 and Figure 5b). Pairwise *F*_ST_ estimates between the 13 groups identified were all significantly different from zero and varied from 0.130 to 0.531, with a mean value of 0.378.

The relationships based on the final groups, their mutual differentiations measured with *F*_ST_ estimate, the distribution of the various races and intermediates in the NJ dendrogram are summarized in Figure 5.

With the exception of Groups 5a and 9a, sorghum genetic diversity appears organized along a limited number of clearly differentiated groups in the West (Guinea-dominated, yet clearly different from one another, Groups 5b and 6), in the South (Kafir-dominated Group 10), in the East (multiracial Groups 1, 2 and 9a) and in the Center (Durra/Bicolor Group 4 and Durra-dominated Group 3), within a background that appears as a broad swarm in central and eastern Africa (weak structure between Groups 7, 8 and 9) with a frequent reference to the Caudatum race component.

### Reference Set of Sorghum

**Figure 6:**
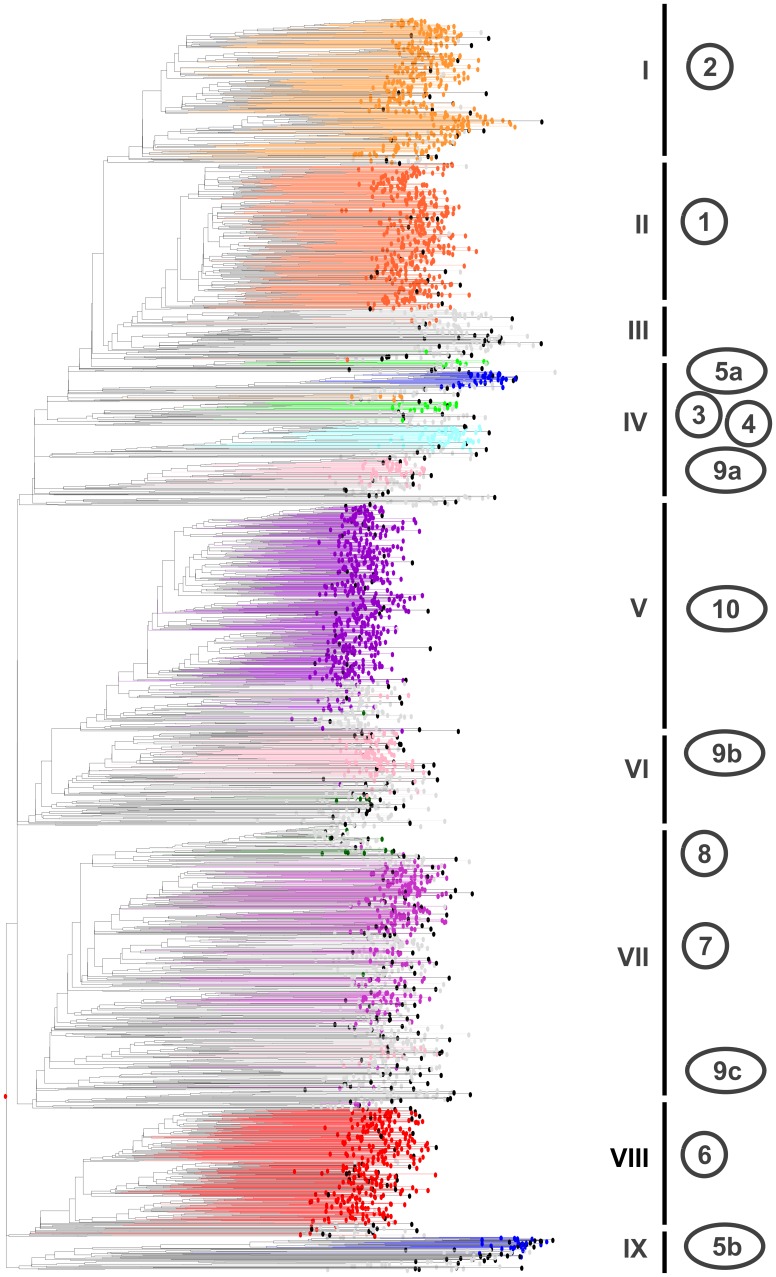
Selected sorghum Reference Set (383 accessions, in black) in relation with hierarchical NJ cluster analysis of 3367 sorghum accessions of a global composite germplasm collection based on allelic data from 41 SSR markers (simple matching distance). Accessions grouped by Bayesian analysis are represented in colors as in Figure 4: Group 1 in orange (C, CB and D from Eastern Asia), Group 2 in light orange (D and B from the Indian subcontinent) Group 3 in light green (D from Eastern Africa), Group 4 in light blue: B and DB from Eastern Africa), Group 5a in dark blue (B accessions assembled from North America) and Group 5b in dark blue (Gma), Group 6 in red (D, DC and G from Western Africa), Group 7 in light purple (C from Central and Eastern Africa), Group 8 in dark green (C and GC from Southern Africa), Group 9a in pink (G from the Indian subcontinent and East Asia), Group 9b in pink (G from Southern Africa), Group 9c in pink (C from Eastern Africa), and Group 10 in purple (GC, K and KC from Southern Africa). Unassigned accessions are presented in grey.

A core reference set with 383 accessions was selected to capture the global genetic diversity of sorghum (Table 3). It includes 332 landraces, 28 breeding lines and 23 wild/weedy accessions, all five cultivated basic races, the 10 intermediate races and accessions of all different geographic origins except South America. It represents the global genetic diversity present in sorghum GCGC (Figure 6). This sorghum reference set captured 78.3% (613 alleles) of the SSR alleles detected in the GCGC, with an average of 14.9 alleles per SSR primer pair (Table 3), comparable to standardized allelic richness of the GCGC. For markers *mSbCIR*306 and *Xisep*0310, all alleles (5 and 10 alleles, respectively) detected in the GCGC were captured in the reference set. Average gene diversity (0.71) in the reference set is slightly larger than for the GCGC. Clustering of accessions in the reference set follows the pattern of race within geographic origin described above for the GCGC. In the case of Gma sub-race, 11 of 12 accessions included in the global composite germplasm collection (all from western Africa) were captured in the reference set.

## Discussion

Maintenance and characterization of large germplasm collections is a huge task. Knowledge of the characteristics of the materials is essential for their efficient management. Both genetic and morpho-agronomical characterizations are required for breeders to better understand and use the available genetic resources. It increases the efficiency of selection of more diverse, adapted, germplasm parents in crop improvement programs. To serve as an entry point to large collections, representative subsets (often referred to as core or minicore collections) provide an economically and logistically attractive option for both gene banks and the breeding programs they serve. However, it is very important that such core collections represent the full range of diversity available at the time of the study. In this context, we used SSR markers to ascertain the population structure of a very large set of sorghum germplasm, in the framework of an international project (the Generation Challenge Programme), consisting of accessions assumed to be representative of global germplasm available for improvement of this crop. This set was used to fine-tune and complete previous knowledge on the evolutionary history and domestication pattern of sorghum. Using this information, a representative subset of this collection was chosen, of a more convenient size for detailed characterization of traits of economic importance to plant breeding programs and for the assessment of allelic diversity in genes associated with variation in such traits.

### A Species-wide Scan Assessment of Neutral Genetic Diversity

#### Breadth of variation

To our knowledge, this is the largest study undertaken in a systematic way for exploring genetic diversity in global crop germplasm. The broad plant material coverage resulted in larger allele numbers (average of 19.2 alleles per locus) and higher diversity parameters than in most previous studies ([48], [49], [50], [51], [32]). It was comparable to the features reported in a focus study on Niger by [30], [52]. The same is true when considering each race separately.

The mean observed heterozygosity (*Ho*) was 0.037, indicating that most markers used detected only one allele per accession, and that the accessions are highly inbred, as expected for accessions of a largely self-pollinated species maintained in collections by enforced selfing. This comparison is notably different when using samples directly derived from landraces, e.g. 0.11 in a Cameroon village [29] or 0.09 in a mix of Guinea race accessions [35].

#### Relevance and distribution of taxonomic components

The high level of genetic diversity in sorghum is thought to be due to multiple origins for domesticated sorghum, intermating between products of these independent domestication events, and continued gene flow between wild and cultivated sorghums [53]. In this study we found substantial evidence of sorghum population structure based on geographic origin and race within geographic origin. This is congruent with previous studies with RFLP markers [11], SSRs [30], SSRs and SNPs [54], and also with recently developed DArT markers ([55], [22]). Yet the structure we observed led us to propose a schematic representation of population structure in sorghum (Figure 5). The periphery harbors types that are more clearly differentiated and more homogeneous. The center harbors more diverse types, with many more intermediates and a concentration of wild types that appear related to several cultivated forms. Among the cultivated accessions, there is hardly any coincidence between a race and a group based on markers, with the single exception of Kafir in Southern Africa. The “races” might better be referred to as morphotypes, or at least consider that races could encompass different morphotypes.

The 68 wild and weedy accessions presented the highest gene diversity and private allele numbers. The majority of wild/weedy sorghum felled in the periphery of Group 5b, but as previously discussed, they were not definitely assigned. The other wild and weedy accessions were distributed, yet on long branches (see Figure S3), in three other dendrogram sectors predominated by cultivated accessions. This contrasts with Aldrich and Doebley's (1992) results [12], who found a clear separation between the two compartments using RFLP markers, as well as Casa et al. 2005 [50] who confirmed this fact with SSR markers. Clearly, the exploration of diversity in a broader representation of wild sorghum is necessary. One can retain yet the broad distribution of wild and weedy accessions throughout the cultivated sorghum diversity patterns, which adds evidence to a corpus of results (including [9], [56], [57], [58], [31]) that suggests that there is considerable exchange of genetic material (gene-flow) between cultivated and wild accessions.

#### A global interpretation of sorghum genetic diversity

Altogether, the geographical pattern of differentiation, the limited congruence between marker-based classifications, the racial classification based on morpho-agronomic traits and the likely occurrence of profuse gene flow advocate for a diversity pattern largely determined by 1) geographical radiation in various directions from the center of origin, with both differential drift among lineages and possibly novel variation selected along the process, 2) common gene exchange among landraces and local wild types, ensuring population dynamics, and 3) selection for race-related trait associations responsible for phenotypic convergence between genetically differentiated sub-populations. Germplasm introduction explains the diversity of the materials contributed to the sorghum GCGC from North America, whereas loss of alleles due to drift appears to have contributed to the reduced diversity observed among samples from India and East Asia compared to those from Africa, with the latter contributing to the observed groupings.

In this scenario, it is likely that the genes that underlie the morphological differences between the most typical morphotypes are few in number and deploying visible polymorphism across geographically differentiated groups. This scenario will be testable when whole-genome genotyping is available in sorghum and may reveal footprints for natural and anthropogenic selection along the genome.

### Community Resources

#### Data

Data generated in the present study was deposited in the GCP central registry (http://generationcp.org/research/research-themes/crop-information-systems, using Sorghum as a ‘crop’ filter, file G2005-01c_Sb_3393accX41SSR_V2.xlsx) and is accessible to the global community. They come in addition to passport data that are available in the germplasm banks and occasional evaluation data that may have been produced as part of searches for donors of specific traits to be used in breeding programs. The data can serve as a reference since it was obtained with an easily accessible kit of markers [24] that can be used on any new material for comparison.

#### Reference set of sorghum

We used the marker data and population structure of the sorghum GCGC from this study to identify a much smaller representative subset of accessions, called the ‘Reference Set’. This Reference Set provides an entry point to sorghum germplasm globally, to identify geographic regions and racial subgroups from which sorghum accessions exhibiting interesting variability in a particular trait can be found. The general value of an internationally agreed set of representative germplasm to serve as a common reference for focussing characterization has been highlighted elsewhere [59].

This proposed Reference Set consists of 383 accessions, includes important germplasm lines used in crop breeding programs, wild accessions and a mini-core collection of genetically diverse accessions for which considerable phenotypic data is already available. Five basic morphological types, ten intermediate ones and wild/weedy accessions from nearly all geographic origins were captured in this sorghum Reference Set. This set represents most of the genetic diversity present in the GCP sorghum Global Composite Germplasm Collection, with all assignation groups and clusters represented. It has a population structure similar to that discussed above for the sorghum GCGC, yet with less redundancy in highly populated narrow clusters. Compared to previously described subsets ([5], used e.g. in [54]) which include converted lines with photoperiod-insensitivity and dwarfing genes, this reference set includes all types of material, enabling breeding choice in Africa. Besides, it also includes wild samples, is more balanced in terms of initial racial classification (more Guinea and less Caudatum in proportion), and represents all geographical origins (and correlatively to racial belonging, represents best West Africa). Seeds are maintained by ICRISAT and available upon request. All passport data published in the System Wide Information Network on Genetic Resources (SINGER), including Sorghum, are available in Genesys (http://www.genesys-pgr.org/), which aims at being the global information system on the germplasm held ex situ.

#### Perspectives

The core reference set is expected to stimulate links among sorghum scientists. The data have been analysed with several methods, which provide marker-based keys to germplasm classification and are meant to serve as a reference. Any new material can easily be compared to this reference; these markers are easily applicable for local studies with local questions in local laboratories, and yielding results that are comparable to other studies performed elsewhere, thanks to the use of a common kit of markers and standards.

This will be very useful for identifying germplasm action priorities, for enriching global collections if novel types are uncovered or for broadening the basis of a given breeding program.

Having the data available for the whole GCGC for 41 SSR loci provides a considerable backup for mining germplasm diversity. Molecular data can serve for complementing reference materials with additional germplasm targeted towards particular applications depending on the operational constraints, the biological constraints (e.g. phenology) and statistical power. Typically SSR data can be used to adjust a sample to a target size with the view to minimizing population structure in order to maximize resolution power in a given association analysis; the Maximum Length Subtree function of the DARwin software can help do this easily, quickly and rigorously [43]. This dynamics will also enable adjusting the reference set by making it inclusive of newly characterized diversity.

In the long term, helping a global community to focus on similar materials for all sorts of biological investigations will help accumulate and compile data in order to develop better biological understanding of sorghum, and of plant biology thanks to sorghum.

## Supporting Information

Figure S1 **Scree plot of the factorial analysis. Proportion of variance for each component, sorted in decreasing order of variance.**
(PDF)

Figure S2 **Graphical method (as in Evanno et al. 2005) allowing detection of the number of groups K (output of CLUMPP process). L(K) for each K. Rate of change of the likelihood distribution (mean ± SD) calculated as L′(K) = L(K) – L(K –1).** Absolute values of the second order rate of change of the likelihood distribution (mean ± SD) calculated according to the formula: |L′′(K)| = |L′(K +1) – L′(K)|. ΔK calculated as ΔK = m|L′′(K)|/s[L(K)].
(EPS)

Figure S3 **Hierarchical NJ cluster analysis of sorghum accessions of a global composite germplasm collection based on allelic data from 41 SSR markers (simple matching distance).** All accessions except Gma are presented. Accessions grouped by Bayesian analysis are represented in colors as in figure 4: Group 1 in orange (C, CB and D from Eastern Asia), Group 2 in light orange (D and B from the Indian subcontinent) Group 3 in light green (D from Eastern Africa), Group 4 in light blue: B and DB from Eastern Africa), Group 5a in dark blue (B accessions assembled from North America), Group 6 in red (D, DC and G from Western Africa), Group 7 in light purple (C from Central and Eastern Africa), Group 8 in dark green (C and GC from Southern Africa), Group 9a in pink (G from the Indian subcontinent and East Asia), Group 9b in pink (G from Southern Africa), Group 9c in pink (C from Eastern Africa), and Group 10 in purple (GC, K and KC from Southern Africa). Unassigned accessions are presented in grey. Wild accessions are presented in black.
(EPS)

Table S1 **List of accessions comprising the sorghum global composite germplasm (GCCG) collection and characterization.** It includes each accession number, institution originally providing the material, biological status, species, subspecies, race, geographic origin (country), geographic origin (continent), subgroup assignation (K value and Cluster), included or not in the Reference Set. Countries are given in standard ISO 3166-1 alpha-3 codes. Continents are AUS (Australia), CAfrica (Central Africa), EAfrica (Eastern Africa), EAsia (Eastern Asia), Europe, IND (India), MedB (Mediterranean Basin), MidE (Middle East), NAmerica (Northern America), SAfrica (Southern Africa), SAmerica (Southern America), WAfrica (Western Africa). Subgroup assignations correspond to Structure K values combined with Cluster assignations. When an accession is included in the Reference Set, last column includes a “one”, on the contrary a “zero”.
(TXT)

Table S2 **Pairwise** ***F*** **_ST_ differentiations between groups as identified in** **Figure 4** **.**
(TXT)
