# Supplementary material for: Massive Sorghum Collection Genotyped with SSR Markers to Enhance Use of Global Genetic Resources
Source: PLoS One. 2013 Apr 2;8(4):e59714. doi: 10.1371/journal.pone.0059714 (PMC3614975; doi:10.1371/journal.pone.0059714)

File SI2. Scree Plot of the factorial analysis. Proportion of variance for each component, sorted in decreasing order of variance.

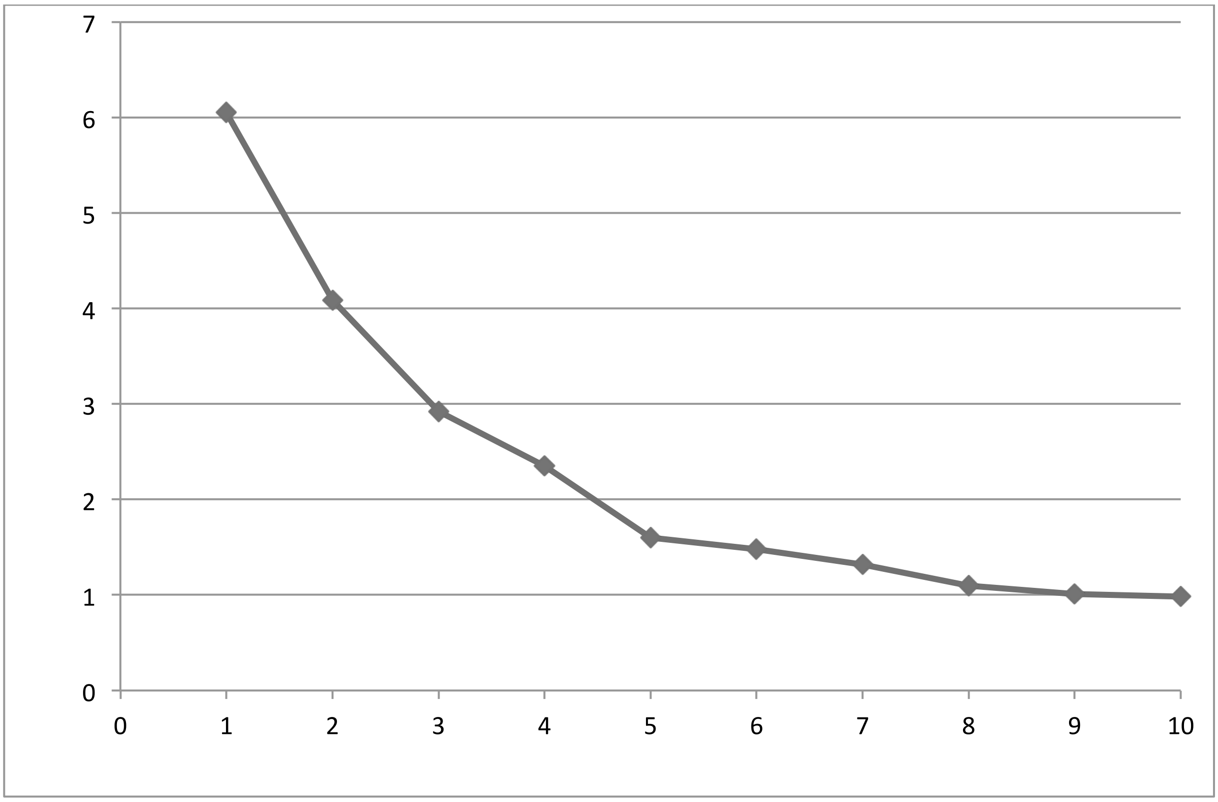

Supplement: Figure S1 — Scree plot of the factorial analysis. Proportion of variance for each component, sorted in decreasing order of variance. (PDF) [file pone.0059714.s001.pdf]
